# A Rank-Ordered Marginal Filter for Deinterlacing

**DOI:** 10.3390/s130303056

**Published:** 2013-03-04

**Authors:** Gwanggil Jeon, Marco Anisetti, Seok Hoon Kang

**Affiliations:** 1 Department of Embedded Systems Engineering, Incheon National University, 12-1 Songdo-dong, Yeonsu-gu, Incheon 406-772, Korea; E-Mail: gjeon@incheon.ac.kr; 2 Dipartimento di Informatica, Universita degli Studi di Milano, via Bramante 65 - 26013 Crema (CR), Italy; E-Mail: marco.anisetti@unimi.it

**Keywords:** rank-ordered filter, adaptive marginal filter, deinterlacing, format conversion

## Abstract

This paper proposes a new interpolation filter for deinterlacing, which is achieved by enhancing the edge preserving ability of the conventional edge-based line average methods. This filter consists of three steps: pre-processing step, fuzzy metric-based weight assignation step, and rank-ordered marginal filter step. The proposed method is able to interpolate the missing lines without introducing annoying articles. Simulation results show that the images filtered with the proposed algorithm restrain less annoying pixels than the ones acquired by other methods.

## 1. Introduction

Interlaced scanning has been advanced from the early days of TV and still adopted for SDTV and 1080i HDTV broadcast standards [1]. However, nearly all late model flat panel displays (LCD, PDP, *etc.*) use progressive scanning formats. For these display devices, an entering interlaced video signal has to be transformed to a progressive one, and thus a scanning format conversion that gives compatibility between various video formats is required [2]. The super-resolution (SR) is a class of techniques that enhance the resolution of an imaging system [3–8]. The deinterlacing only considers vertical direction, while SR considers both of vertical and horizontal directions. Thus, the intra-field deinterlacing is a special case of SR.

Many deinterlacing methods have been proposed, including spatial methods [9–13] and motion-based methods [14]. Although motion-based methods yield better subjective quality than spatial methods, they require reliable motion models and the estimated trajectories must be sufficiently proper, which generally causes excessive computational complexity On the other hand, spatial methods have lower computational complexity since they only demand the current frame, making them more suitable for real-time applications. Therefore, in this paper, we focus on the spatial method.

Among spatial approaches, deinterlacing based on edge direction is the most outstanding and broadly adopted method. These methods calculate edge information first and then decide edge direction to utilize appropriate pixels for interpolation. Thus the edge information calculation and edge direction decision are the key steps. However, conventional methods have yielded poor performance when edge direction is not credible.

To shorten this issue, we propose a deinterlacing algorithm using rank-ordered fuzzy metric approach to reduce artifacts in deinterlaced images. In our approach, the missing lines are calculated by weight obtained using fuzzy metric (*FM*) from the existing neighbor pixels. The local *FM* infers the weight of the edge information. Thus, we deinterlace the interlaced signal without calculating edge directions as the traditional approaches do. After that, the rank-ordered differences statistic introduced in [15] is accommodated to the fuzzy context utilizing the introduced *FM*.

The paper is arranged as follows. Section 2 introduces *FM* used in the weight assignation step. After that, the proposed filtering technique is described. Section 3 shows simulation results including performance comparison and computational complexity. Finally, conclusions are drawn in Section 4.

## 2. Proposed Method

### 2.1. Fuzzy Metric for Weight Assignment

A stationary *FM*, on a set *S*, is a fuzzy set of *S* × *S* satisfying the following conditions for all *p*,*q*,*r* ∈ *S* [15]:

*Rule*_1_: *FM_S_*(*p*,*q*,*t*) > 0;
*Rule*_2_: *FM_S_*(*p*, *q*, *t*) = 1 if and only if *p* = *q*;
*Rule*_3_: *FM_S_*(*p*,*q*,*t*) = *FM_S_*(*q*,*p*,*t*);
*Rule*_4_: *FM_S_*(*p*, *q*, *t*) ≥ *FM_S_*(*q*, *r*, *u*) * *FM_S_*(*p*, *r*,*t* + *u*);

where *Rule_#_* is a *FM* of rule number #, * is a continuous *t*-norm, *FM_s_*(*p*, *q*, *t*) stands for the degree of nearness between p and q according to *Rule*_2_, *FM_s_*(*p*, *q*, *t*) is close to 0 when *p* is far from *q*. Let *S* be the set {0,1,…, 255}, then, the function *FM : S* × *S* → [0,1] given by

(1) $$FM_{S}(p,q)={\left(\frac{\text{min}(p,q)+b}{\text{max}(p,q)+b}\right)}^{a}$$

where *b* is a small positive value for preventing *max*(*p*, *q*) = 0 singularity. As the difference between the components *p* and *q* become bigger, the value of *FM_s_* falls quickly. Thus, we assume *FM_s_*(*p*, *q*) is the fuzzy distance between the image components *p* and *q*. Clearly, *FM* is *F*-founded and it meets

(2) $$0\leq \frac{b}{I_{\text{max}}+b}\leq FM_{S}(p,q)\leq 1$$

for all *p*,*q* ∈ *S*, *I_ma_x* is maximum pixel intensity, and *I_max_* = 255 in this paper.

### 2.2. Deinterlacing Implementation

The proposed filter consists of three steps: (1) pre-processing step, (2) *FM*-based weight assignation step, and (3) rank-ordered marginal filter step. To begin with, we conduct interpolation with three missing pixels at location (−1, 0), (0, 0), and (1, 0), with vertical six-tap filters. After that, we evaluate *FM* degree using the introduced *FM* equation. The obtained *FM* degree is used for assigning weights. Finally, the missing pixel is calculated using the rank-ordered marginal filtering (ROMF) scheme.

Let us assume that *I* is an image and *I*(*_c_*,*_r_*) is the pixel intensity at a position of (*c*, *r*), *c* is column number and *r* is raw number, and *I*_(0,0)_ is the centered missing pixel to be processed. We denote *W* as a filtering window centered on the pixel under processing of size *N*×*N*,*N* = 3,5,7,…, which contains *n* = *N*^2^ pixels. The pixels in *W* are symbolized as *I*(*_c_*,*_r_*), and *c*, *r* = −1, 0,1 for *N* = 3 case.

**Figure 1.**
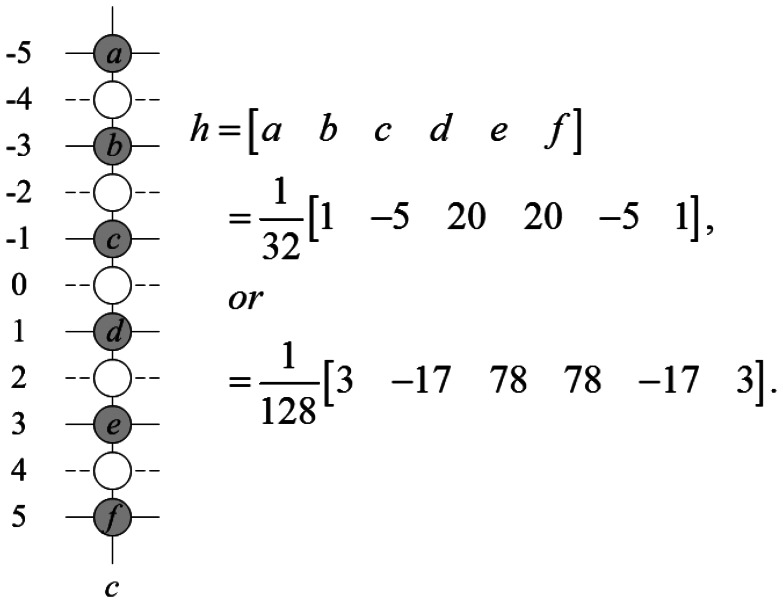
The pixel positions with filter coefficients.

The first step of the ROMF method is vertical six-tap filter (STF). This fixed coefficient six-tap Wiener filter is widely used to estimate the sub-pixels in video codec, such as MPEG-4, H.264/AVC, and some deinterlacing methods [16]. The coefficients of this filter can be different such as *h* = [1, −5, 20, 20, −5, 1]/32 or *h* = [3, −17, 78, 78, −17, 3]/128. In this paper, we chose the previous one for our system under the assumption that *h* can calculate missing lines in the sub-pixel position properly. The missing pixels at (*c*, 0) position, *c* = −1,0,1, are estimated using the adjacent pixels at (*c*, −5), (*c*, −3), (*c*, −1), (*c*, 1), (*c*, 3), and (*c*,5), and we denote them as *I*(_c_,_−5_), *I*_(_*_c_*_,−3)_, *I*_(c,−1)_, *I*_(_*_c_*_,1)_, *I*_(_*_c_*_,3)_, and *I*_(_*_c_*_,5)_, respectively. To interpolate the pixel more precisely, we must adapt the filter to accommodate the new interpolation condition. Now, three pixels in the missing line $I_{\left(-1,0\right)}^{\text{STF}}$, $I_{\left(0,0\right)}^{\text{STF}}$ and $I_{\left(1,0\right)}^{\text{STF}}$ are approximately deinterlaced applying Equation (3); however, they are not the same with the original missing pixel. Figure 1 shows the pixel positions with filter coefficients.

(3) $$I_{\left(c,0\right)}^{\text{STF}}=h(1)I_{\left(c,-5\right)}+h(2)I_{\left(c,-3\right)}+h(3)I_{\left(c,-1\right)}+h(4)I_{\left(c,1\right)}+h(5)I_{\left(c,3\right)}+h(6)I_{\left(c,5\right)}$$

For the ROMF, eight neighboring pixels, *I*_(−1,−1)_, *I*_(0,−1)_, *I*_(1,−1)_, $I_{\left(-1,0\right)}^{\text{STF}}$, $I_{\left(1,0\right)}^{\text{STF}}$, *I*_(−1,1)_, *I*_(0,1)_ and *I*_(1,1)_ are employed to deinterlace the missing center pixel at (0,0). In this paper, we take *FM_S_* as the distance function (note, however, that any other function such as Euclidean distance could be used). Therefore, the distance between two pixels $I_{\left(0,0\right)}^{\text{STF}}$ and *I*_(_*_c_*,*_r_*_)_ is symbolized as *FM_S_* ( $I_{\left(0,0\right)}^{\text{STF}}$,*I*_(_*_c_*,*_r_*_)_). We denote *W̅* the set of neighbors of $I_{\left(0,0\right)}^{\text{STF}}$, that is, $\bar{W}=W-I_{\left(0,0\right)}^{\text{STF}}$.

The second step is to calculate eight *FM* using *FM_s_*( $I_{\left(0,0\right)}^{\text{STF}}$, *I*_(_*_c_*,*_r_*_)_) where *I*_(_*_c_*,*_r_*_)_ ∈ *W̅*. The proposed deinterlacing solves the problem by looking for the most robust *I*_(_*_c_*,*_r_*_)_ pixel. To compute ROMF, the distance *FM_S_*( $I_{\left(0,0\right)}^{\text{STF}}$,*I*_(_*_c_*,*_r_*_)_) are rearranged in an ascending order so that a group of non-negative real values *χ_m_*, where fixed a positive integer *m ≤ n* − 1, are obtained. Note that *χ_m_* is not always different: *χ*_1_ ≤ *χ*_2_ ≤ … ≤ *χ_m_* ≤ … *χ_n_*_−1_. Generally speaking, *χ_j_* is the *j^th^* smallest *FM_S_*( $I_{\left(0,0\right)}^{\text{STF}}$,*I*_(_*_c_*,*_r_*_)_) value, and its associated *I*_(_*_c_*,*_r_*_)_ is denoted as *I_χj_*. Finally, the proposed ROMF calculates the missing pixel $I_{\left(0,0\right)}^{\text{ROMF}}$:

(4) $$I_{\left(0,0\right)}^{\text{ROMF}}=\frac{1}{2}I_{\left(0,0\right)}^{\text{STF}}+\frac{1}{2}\frac{\sum_{j=-1}^{m}\chi_{j}\cdot I_{\chi j}}{\sum_{j=-1}^{m}\chi_{j}}$$

where *χ_j_* is assumed to be a weight factor. It can be observed from Equations (1) and (4) that, when *I_χj_* and $I_{\left(0,0\right)}^{\text{STF}}$ have similar values, the weight factor *χ_j_* becomes large. On the other hand, when the difference between *I_χj_* and $I_{\left(0,0\right)}^{\text{STF}}$ are large, *χ_j_* becomes smaller. Thus, the missing line is deinterlaced based on the similarity among their neighbor pixels, *I_χj_*, with allocated weights based on the *FM*.

## 3. Simulation Results

To evaluate the performance of the proposed algorithm, we present the simulation results in this section. We considered twenty images and video sequences as the dataset, which are shown in Table 1. The ten images starting with “A” to “G” are the test images, and the others (images starting with “K” to “Z”) are the training images.

**Figure 2.**
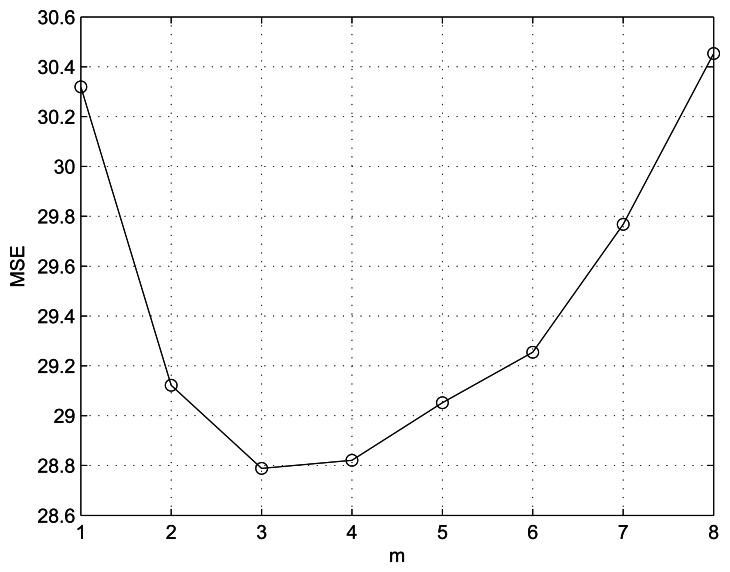
Performance of the proposed method in terms of MSE as a value of *m* under the condition of *b* = 1, for 1 ≤ *a* ≤ 15.

**Figure 3.**
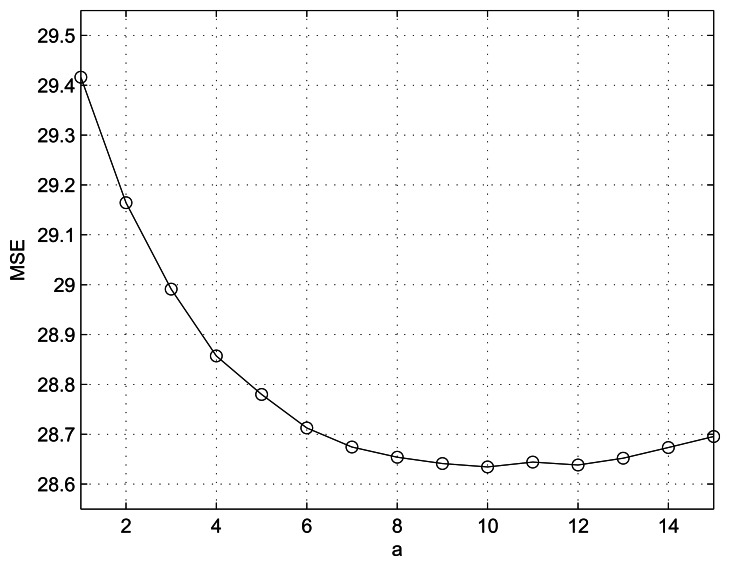
Performance of the proposed method in terms of MSE as a value of *a* under the condition of *b* = 1 and *m* = 3.

We conducted simulation using MATLAB with an Intel(R) Core(TM) i5 CPU M460 @ 2.53 GHz processor. We compared the proposed method with MELA [9], LABI [10], FEPD [11], MCAD [12] and LSMD [13] methods. Note that the designed filter parameters *a* and *b* and the number of considered neighbor pixels *m* play crucial roles, making it important to set them appropriately. One assumption is that, as we mentioned in Section 2, parameter *b* is a small positive value for avoiding *max*(*p*,*q*) = 0 singularity. Thus we gave *b*=1, which is the smallest intensity step. Figure 2 shows the average MSE performance of the proposed method according to various *m* values under the condition of *b* = 1 and 1 ≤ *a* ≤ 15. From Figure 2, m = 3 is determined to give the least MSE. Another parameter *a* = 10 is determined under the condition of *b* = 1 and *m* = 3, as shown in Figure 3.

The PSNR metric in decibels (dB) was selected to evaluate the performance. Table 2 shows the comparison results of the PSNR performance of the proposed method to the benchmarks. After the experiments, it is obvious that the proposed method outperforms other methods by 0.959 (MELA), 1.199 (LABI), 2.414 (FEPD), 1.541 (MCAD), and 1.377 (LSMD) dB in terms of average PSNR. For Akiyo and Bus image, MELA showed a better PSNR performance of 0.107 dB and 0.035 dB. However, the proposed method showed the best PSNR performance for the other images.

Table 3 shows the CPU time per image. As we can see, the proposed method has more complexity than MELA. However, the proposed technique reduces the average CPU time up to 93.74%, 96.89%, 95.57%, and 79.52% when compared with LABI, FEPD, MCAD, and LSMD, respectively.

**Figure 4.**
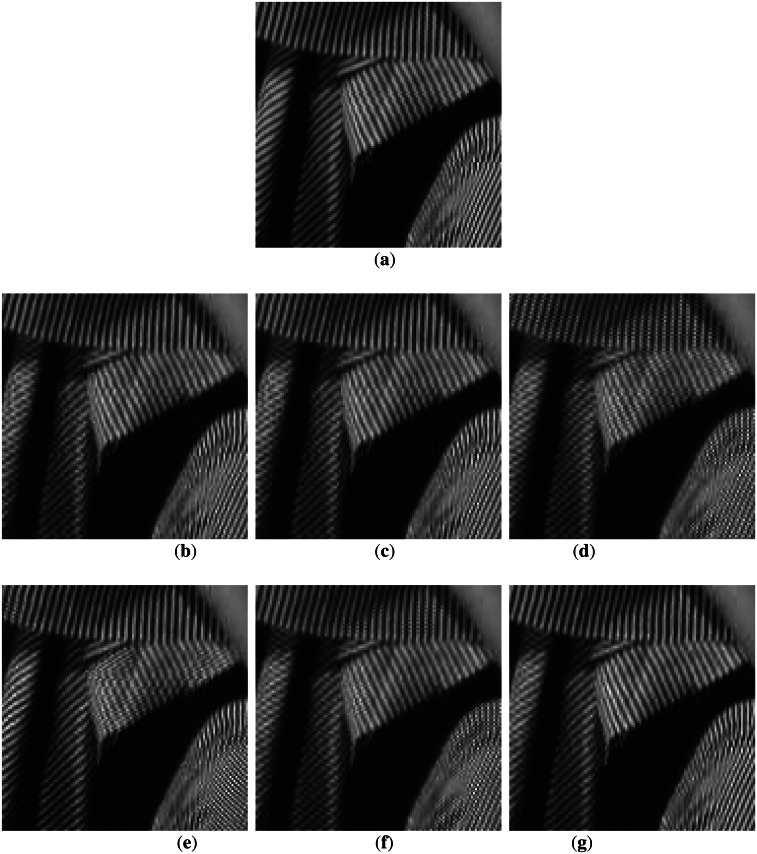
Comparison of subjective qualities in Barbara image: (**a**) original Barbara; (**b**) MELA; (**c**) LABI; (**d**) FEPD; (**e**) MCAD; (**f**) LSMD; and (**g**) ROMF.

**Figure 5.**
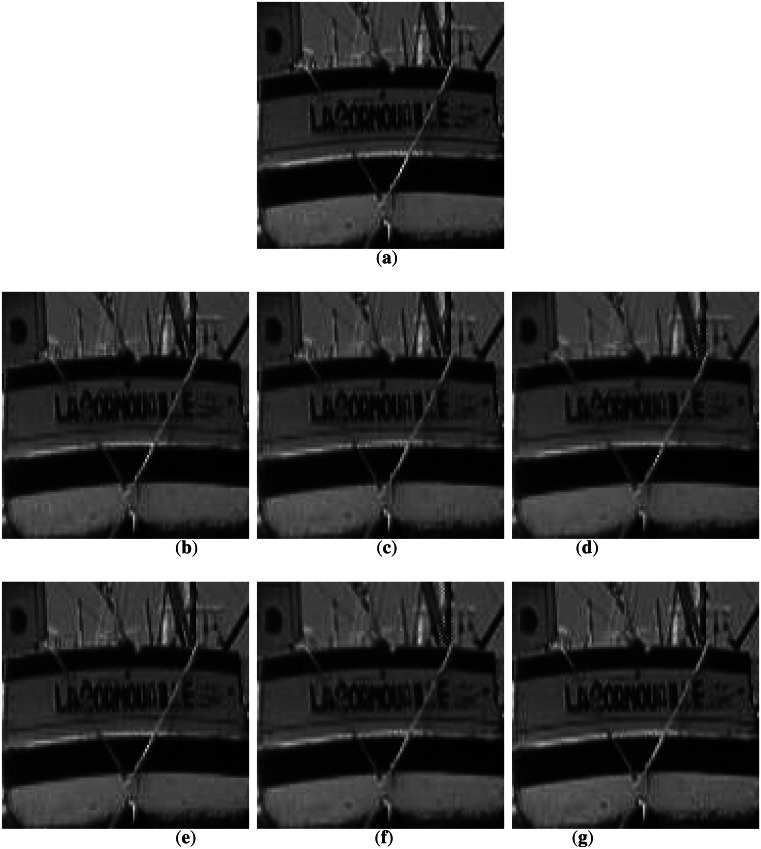
Comparison of subjective qualities in Boat image: (**a**) original Boat; (**b**) MELA; (**c**) LABI; (**d**) FEPD; (**e**) MCAD; (**f**) LSMD; and (**g**) ROMF.

The Barbara image in Figure 4 has many low and high-angle directions that the previous methods may miss. Figure 4(a–g) shows poor performance because only a limited number of edge directions were utilized, which does not compensate for inaccurate edge information. Figure 4(h,i) shows better results than the other conventional methods. However, the diagonal edge reconstruction is not sufficient. The proposed method, however, performs well for this case as shown in Figure 4(j). Figure 5 shows the results for the Boat image. The result for this image also shows that the proposed method is superior to other methods.

## 4. Conclusions

This paper presented an effective spatial deinterlacing method, which is achieved by improving the edge preserving ability of the conventional edge-based line average method. This filter consists of three steps: pre-determined six-tap filter based pre-processing step, *FM*-based weight assignation step, and rank-ordered marginal filter step. The experimental results indicated that ROMF has achieved these two goals and has promising performance subjectively and objectively. Meanwhile, ROMF has merits of low complexity for real-time application.

## Figures and Tables

**Table 1.** Test and training sets classified by alphabetical order.

Test images (I) and video (V) sequences (images starting with “A” to “G”):
Airplane (I), Akiyo (V), Barbara (I), Bluesky (V), Boat (I), Bus (V), City (V), Finger (I), Football (V), Girl (I)
Training images (I) and video (V) sequences (images start with “K” to “Z”):
Kimono (V), Lena (I), Man (I), Milkdrop (I), Mobile (V), News (V), Peppers (I), Raven (V), Toys (V), Zelda (I)

**Table 2.** Comparison of the average PSNR for 10 test images and video sequences among different deinterlacing methods.

	MELA	LABI	FEPD	MCAD	LSMD	ROMF	Ranking
airplane	35.088	35.345	34.385	35.085	35.660	36.084	1
akiyo	40.205	38.841	37.255	39.726	38.149	40.098	2
barbara	32.018	31.930	28.879	25.929	29.414	33.562	1
bluesky	37.900	37.798	37.510	38.107	39.373	39.547	1
boat	35.186	35.277	33.074	35.342	33.762	36.034	1
bus	28.654	28.217	28.104	28.262	28.095	28.619	2
city	31.460	31.497	31.258	31.527	31.656	31.726	1
finger	31.323	31.362	30.679	31.810	32.085	32.946	1
football	35.057	34.475	33.308	35.034	34.763	35.791	1
girl	41.793	41.535	39.676	42.038	41.545	43.861	1
avg.	34.868	34.628	33.413	34.286	34.450	35.827	1

**Table 3.** Comparison of the average CPU time for 10 test images and video sequences among different deinterlacing methods.

	MELA	LABI	FEPD	MCAD	LSMD	ROMF	Ranking
airplane	0.547	14.698	28.491	22.547	4.286	1.231	4
akiyo	0.207	5.694	10.947	7.861	1.838	0.596	4
barbara	0.490	14.154	29.527	20.609	4.409	0.787	4
bluesky	3.076	123.768	228.331	159.369	34.895	6.067	4
boat	0.470	13.490	28.727	20.191	4.076	1.107	4
bus	0.164	5.406	10.919	8.846	2.304	0.703	4
city	1.358	48.484	105.553	73.752	16.502	2.590	4
finger	0.397	12.349	29.005	20.315	4.036	1.057	4
football	0.203	5.057	12.676	7.755	1.668	0.794	4
girl	0.433	12.305	30.801	19.886	4.070	1.065	4
avg.	0.734	25.54	51.498	36.113	7.809	1.599	4
